# Coupling between Theta Oscillations and Cognitive Control Network during Cross-Modal Visual and Auditory Attention: Supramodal vs Modality-Specific Mechanisms

**DOI:** 10.1371/journal.pone.0158465

**Published:** 2016-07-08

**Authors:** Wuyi Wang, Shivakumar Viswanathan, Taraz Lee, Scott T. Grafton

**Affiliations:** 1 Institute for Collaborative Biotechnologies and the Department of Psychological & Brain Sciences, University of California Santa Barbara, Santa Barbara, California, United States of America; 2 Department of Neurology, University Hospital of Cologne, Cologne, Germany; Centre de Neuroscience Cognitive, FRANCE

## Abstract

Cortical theta band oscillations (4–8 Hz) in EEG signals have been shown to be important for a variety of different cognitive control operations in visual attention paradigms. However the synchronization source of these signals as defined by fMRI BOLD activity and the extent to which theta oscillations play a role in multimodal attention remains unknown. Here we investigated the extent to which cross-modal visual and auditory attention impacts theta oscillations. Using a simultaneous EEG-fMRI paradigm, healthy human participants performed an attentional vigilance task with six cross-modal conditions using naturalistic stimuli. To assess supramodal mechanisms, modulation of theta oscillation amplitude for attention to either visual or auditory stimuli was correlated with BOLD activity by conjunction analysis. Negative correlation was localized to cortical regions associated with the default mode network and positively with ventral premotor areas. Modality-associated attention to visual stimuli was marked by a positive correlation of theta and BOLD activity in fronto-parietal area that was not observed in the auditory condition. A positive correlation of theta and BOLD activity was observed in auditory cortex, while a negative correlation of theta and BOLD activity was observed in visual cortex during auditory attention. The data support a supramodal interaction of theta activity with of DMN function, and modality-associated processes within fronto-parietal networks related to top-down theta related cognitive control in cross-modal visual attention. On the other hand, in sensory cortices there are opposing effects of theta activity during cross-modal auditory attention.

## Introduction

Our environment exposes us to a constant barrage of independent simultaneous sensory events across multiple sensing modalities. In order to make sense of this multitude of information, it is often necessary to ignore irrelevant sensory information from particular sensory modalities or spatial locations while selectively enhancing the processing of other streams of information through the use of cross-modal attention control [1,2]. Achieving cross-modal attention control requires both modality-specific mechanisms as well as general top-down (i.e., supramodal) cognitive control [3,4]. A fundamental question is whether top-down mechanisms are required to enhance processing in specific sensory modalities and if these can be demonstrated by electrophysiological means in humans [5,6]. Cross-modal visual and auditory attention and their underlying neural characteristics have been extensively studied. Recent studies show that the modulation of both amplitude and phase of EEG oscillation could be important mechanisms to enable cross-modal interactions [7–9]. In addition, a recent EEG and fMRI study showed that when there is competition between visual and auditory stimuli, requiring active endogenous control of attention, there is modulation of activity within the sensory cortices, sensorimotor areas and the default mode network (DMN) [10].

It has been hypothesized that cross-modal multisensory control, along with several other forms of cognitive control rely on underlying brain dynamics that are reflected in EEG theta oscillations (4–8 Hz). This low-frequency oscillation, commonly thought to emanate from frontal cortex, is a strong candidate for organizing activities across widely separated cortical regions during cognitive processes [11–13]. Recent work shows that theta oscillations have an important role in top-down control [14,15]. Furthermore, source localization of theta oscillations were found to be situated broadly within regions associated with cognitive control including the frontal cingulate cortex (CC) as well as motor areas, sensory cortex, and the basal ganglia (BG)[11]. Based on these prior studies, we hypothesized that theta modulation would be important for supramodal control of attention. Several studies have proposed that the underlying neural activity producing frontal theta oscillations can facilitate or inhibit other regions by distal synchronization in the theta band [11,16]. These putative local-distal interactions are a challenge to identify from EEG data alone. An alternative approach that we adopt here is to treat the magnitude of EEG theta oscillation as a regressor that can be correlated with simultaneously recorded fMRI BOLD activity. To do this EEG oscillations in a pre-defined frequency band were derived over short blocks of similar trials, (although it is also possible to adopt this approach on a trial by trial basis for suitable experimental designs) [17–21]. This allows for an identification of putative correlates of EEG oscillations assessed over the whole brain. Taking this approach, a previous study showed that BOLD activity within the medial prefrontal and temporal-parietal cortices correlated with theta band amplitude [22]. In another study, there was a negative correlation between frontal theta power and BOLD in DMN during working memory maintenance [19]. In this light, it is possible that theta oscillations play a role in maintaining task performance or possibly limit a switch of cognition to the DMN, which is known to be involved in stimulus independent thought [23].

We adopted a mixed experimental design with cross-modal visual and auditory stimuli analogous to those used previously in the literature [1,24]. Participants were simultaneously presented with both auditory and visual information in several different spatial locations and had to attend to a single modality and a single location until receiving an occasional cue to switch modalities/locations. We used naturalistic pictures and sounds of objects and animals as stimuli to broaden the generalizability of our findings. By including multiple spatial locations to attend to, we could increase task difficulty and identify any confounding interactions between behavioral performance, spatial attention and sensory modality.

As noted above, source localization has linked theta oscillations to the medial frontal cortex and thus, is thought to be related to networks involved in cognitive control [11]. In this study, we extend this view by testing for interactions between theta modulation (measured at Fz and Cz electrodes, overlying medial frontal cortex) and brain activity, as measured by fMRI voxel-wise BOLD activity. If theta power influences a functional system, there should be a correlation between theta amplitude and regional BOLD in that system [17,25]. With our approach, we can assess two potential relationships between theta amplitude and BOLD: a. Supramodal networks—These are areas of significant correlation between theta and BOLD for visual, auditory, and critically, the conjunction of visual and auditory selective attention. That is, no matter what the modality, the supramodal network is engaged. b. Modality specific networks—These are brain areas of significant theta-BOLD correlation that are significant for auditory or visual processing alone. We note that this is not the same as directly comparing task related activity during the two modalities of attention, which gives an indication of the effect of cross-modal attention on sensory processing. Rather, it is the differential influence of theta related activity with underlying circuits supporting either auditory or visual attention.

## Materials and Methods

### Ethical Statement

All subjects provided informed written consent prior to experimental sessions, all procedures were approved by the Human Subject Committee, Office of Research, University of California Santa Barbara. All participants were paid for participating in the experiment.

### Subjects

We collected simultaneous EEG-fMRI and behavioral data from 17 right-handed healthy adult subjects aged 18–26, (7 males) recruited from the local community. Two participants were excluded from further analysis due to unreliable recordings of behavioral data leaving a final sample of 15 participants. All subjects had normal or adjusted-to-normal vision, and no history of psychiatric disorders and neurological diseases.

### Stimuli, Task and Experiment Structure

**Fig 1:**
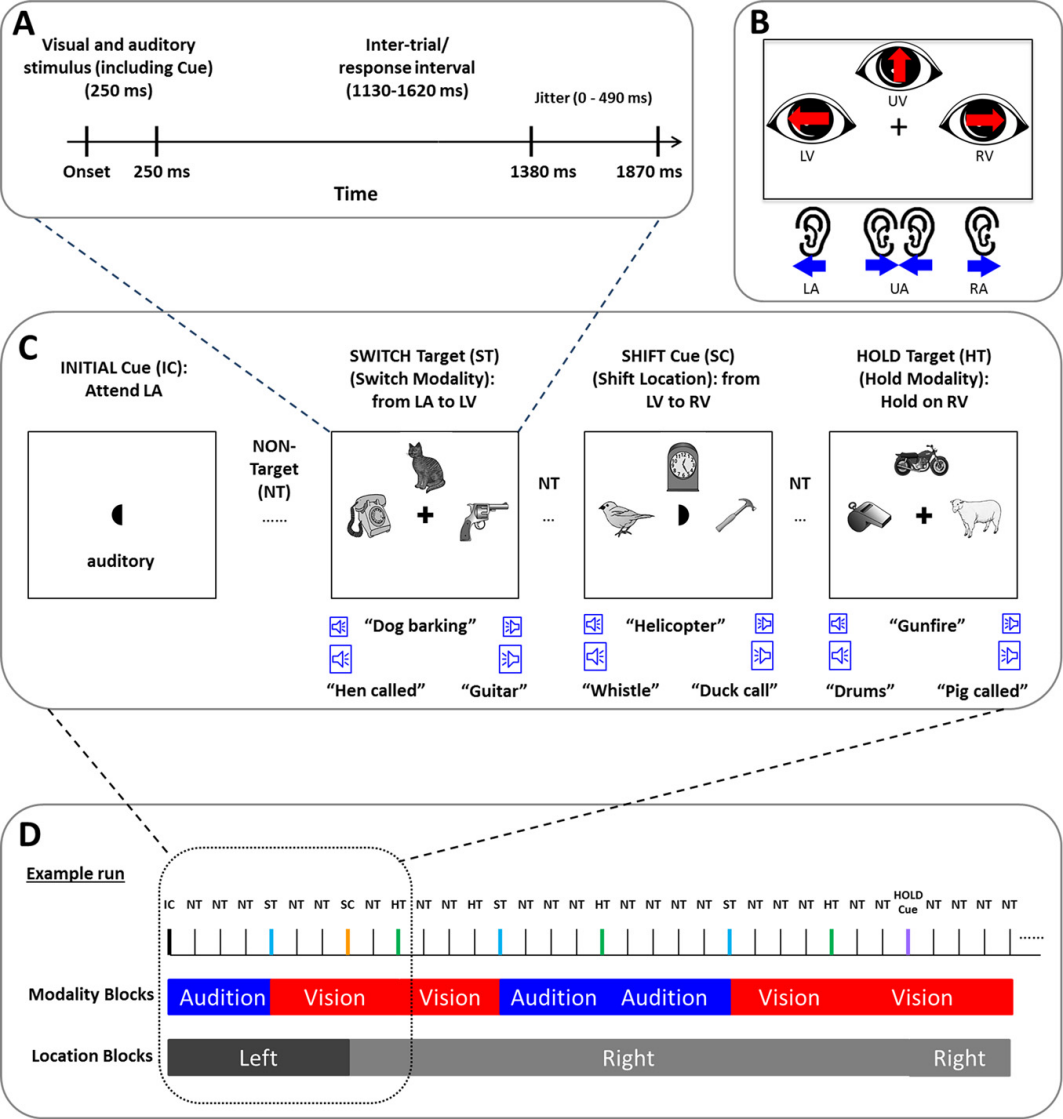
The experimental paradigm. (A) The timing of one trial. (B) On any given trial, the subject was relying on one of six possible states of covert attention. These corresponded to a visual (upper panel) or auditory focus of attention (lower panel). In addition, subjects focused attention to the left, right or upward perceptual space. (C) A structural diagram showing that three stimuli each are presented in the visual and auditory modalities while the subjects also maintain spatial attention to any of three locations. Examples of targets indicating a switch or hold between sensory modalities or moon cues indicating a shift in spatial attention are also shown. (D) An example of a run and the independent blocking structure for object and spatial attention. The individual blocks of the two attended sensory modalities (visual and auditory conditions) as a function of spatial location were analyzed in both EEG and fMRI data analysis.

Subjects were required to detect specific visual or simultaneously presented auditory stimuli. The visual stimuli consisted of 22 pictures of different objects selected from the Snodgrass and Vanderwart set [26]. The pictures included animals, vehicles, instruments, weapons, and tools. All the pictures were converted to grayscale, and scaled to be of the same size and luminance. For each trial, three different stimuli were simultaneously displayed at three locations on the computer monitor (i.e. left, right and up) surrounding a central fixation cross (See Fig 1C). On a subset of trials (see below), at trial onset the central fixation cross was briefly replaced with one of three “crescent moon” shaped cues. The curved side of the moon indicated the appropriate spatial direction of attention (left, right or up) to use for all subsequent trials. Both the visual targets and occasional moon cues were only presented for 250 ms.

A set of 22 auditory stimuli that matched the identity of the visual stimuli were obtained from a free sound website (www.freesound.org). The original sound files were edited to have the same sampling rate and to be exactly 250 msec in duration. For each trial subjects were presented with three auditory stimuli: One for the left ear, one for the right ear and another in both ears. The stimulus presented to both ears was spatially perceived to be in the midline in an upward direction, analogous to the "up" visual position (See Fig 1C). The amplitudes of auditory stimuli were adjusted such that the intensity of a stimulus presented to both ears was 70% of the intensity of a stimulus in one ear.

Participants were asked to detect either of two targets (a chicken or a sheep, counterbalanced across participants) presented in the attended sensory modality (auditory or visual) and in one of the three attended spatial positions (left, right or up). The subject was required to maintain attention in one sensory modality or the other until a target was detected. Similarly, they were required to attend to a single spatial location until a visual moon cue at fixation indicated a change. One of the two targets (chicken/sheep) indicated that the subject should continue to look for targets in the currently attended modality (hold trial). The other target indicated that subjects should switch their focus of attention to the other sensory modality (switch trial). To monitor performance, participants were asked to press a button with the right hand whenever they detected either type of target and to withhold button pressing for all other stimuli. Reaction Time (RT) was defined as the time between target onset and the button response, accuracy was defined as the ratio between correct response trials and all trials, and all other button presses were coded as false alarms (FA). The instructions for directing spatial attention, the central visual moon cues, were presented in random order, and timed to be independent of targets. Thus, for any given trial there were six possible cross-modal attentional states for each subject: Left Visual (LV), Left Auditory (LA), Upper Visual (UV), Upper Auditory (UA), Right Visual (RV) and Right Auditory (RA), as shown in Fig 1B.

The experiment involved two sessions on different days. There was a half-hour training session on the first session, followed by the main experiment on the second session in the fMRI scanner. Both sessions were completed within 3 days of each other. During the training session, participants were first familiarized with all the auditory and visual stimuli that would be presented in the task; and trained to identify and name the target stimuli. Participants practiced the task until they were able to perform the task with greater than 90% accuracy. All participants were prepared for EEG and then positioned in the MRI scanner. All visual stimuli were back-projected into the scanner bore, viewed by a coil-mounted mirror. All auditory stimuli were delivered by an MRI-compatible headphone (3M Peltor Optime l H510A Headband Ear Muffs). At the beginning of each functional MRI run, a prompt was displayed that indicated which of the two modalities (auditory or visual) the participants should attend to and what spatial location to attend to for at the outset of that run. Participants were instructed to maintain eye gaze to the central fixation cross or moon throughout each run. They were verbally reminded to only monitor the relevant sensory modality until they detected the switch target at which point they should monitor the other sensory modality before the start of formal run. The ratio of visual targets and auditory targets was 1:1; the ratio of target and non-target stimuli was 1:3; the ratio of switch targets and hold targets was 1:1 (all trials with switch targets were excluded from following behavioral, EEG and fMRI data analysis); the ratio of stimuli with spatial cues versus stimuli without spatial cues was 1:24; and the ratio of spatial cues indicating the same versus a new spatial direction was 2:1 (all trials with spatial cue were ignored on following EEG and fMRI data analysis). This led to the formation of mini-blocks of consecutive trials of a similar type as defined in Fig 1B. This blocking structure facilitated analysis of fMRI data, while also allowing for EEG analysis of single rapidly presented trials. The order of target stimuli were pseudo-random: The switch targets only occurred in the attended sensory modality, while the hold targets could occur in the attended modality or as lures in the unattended sensory modality. After the stimuli were displayed for 250 msec, there was a waiting period with a pseudorandom duration from 1.13 to 1.62 sec, as shown in Fig 1A. Such a temporal jitter aided in estimating blood oxygenation level dependent (BOLD) signal responses.

Each participant performed a series of practice trials before enter into MR scanner, then 6 full fMRI-EEG runs. Each fMRI run lasted approximately 16 min. In total, there were 3600 trials (600 for each of the 6 attention conditions). An example run is shown in Fig 1D. Participants were encouraged to maintain their focus of attention on the appropriate sensory modality and spatial location even if they missed a target during run. To determine if subjects were attending to the correct sensory modality, motor responses were evaluated in relationship to blocks of consecutive trials of the same expected sensory modality. Only those blocks of trials where correct motor responses were made to switch or hold targets were considered in further analyses. The overall number of blocks of trials that were rejected because of false alarms or missed switch/hold targets was 16%.

### Eye Tracking

To assess visual fixation, each participant was monitored with an EyeLink 1000 eye tracker (www.sr-research.com). This eye tracker records monocular eye positions at 1000 Hz by an MRI compatible camera and automated video processing using the same way as reported on previous study [27]. The experimenters monitored qualitative eye position for each trial and provided feedback when subjects deviated from the central position. Only subjects who maintained visual fixation throughout the study are included in the analysis.

### EEG data acquisition and analyses

EEG data were recorded simultaneously with fMRI data by using a 64 channel MRI-compatible BrainAmp MR Amplify Plus system (Brain Products, Germany, http://www.brainproducts.com), along with a suitable electrode cap (Falk Minow Services, Germany), which is based on the extended 10–20 system and a reference electrode located in between Fz and Cz. Horizontal eye movements were detected by using the electrodes located nearest to the left and right outer canthi respectively (i.e. HEOG and IO) while vertical eye movements and blinks were detected with frontal electrodes FP1 and FP2. In addition, an electrocardiogram (ECG) electrode was fixed on the skin of the back at the level of the participant's heart. Both EEG and ECG signals were sampled at 5 KHz, and the impedances of electrodes were kept below 20 kΩ across the entire recording period.

The EEG signals were analyzed using Brain Vision Analyzer 2.0 (Brain Products, Germany) and EEGLAB 11 (sccn.ucsd.edu/eeglab) in Matlab (The MathWorks) software. MRI gradient switching artifacts were removed via Brain Vision Analyzer. The correction process creates an artifact template for each TR based on scanner triggers, and this template is subtracted from the raw EEG data [28]. Following this correction, the data was down sampled to 250 Hz. The ballistocardiogram artifact was removed via Niazy’s OBS method [29] using the FMRIB plugin for EEGLAB. After BCG correction the data from the ECG electrode was discarded. The ocular and muscular artifacts were removed using AAR 1.3 (Automatic Artifact Removal toolbox) plugin for EEGLAB 11. After ocular and muscular correction, the FP1, FP2, HEOG and IO electrodes were excluded from further analyses. The resulting signal from each electrode in each imaging session was band pass filtered between 0.1 and 35 Hz using the Basic FIR filter available in EEGLAB.

Following this preprocessing, EEG data were analyzed with two approaches: block-wise and trial based frequency analysis. For the block-wise analysis, consecutive trials corresponding to the same attention condition (i.e. LV, LA, RV, RA, UV and UA) were combined as a continuous epoch for which filter analysis was performed. As noted above, we excluded all blocks where participants made at least one wrong response (by either failing to respond to a switch or hold target in the relevant sensory modality or by incorrectly responding to a hold target in non-relevant sensory stream). In order to assess theta band oscillations in each block, the EEG spectrum from 4 to 8 Hz was calculated from average of Fz and Cz electrodes of each subject. The ITC (inter-trial coherence, i.e. event-related phase-locking) and ERSP (event-related spectral perturbation) were calculated on each trial. For each subject, the absolute value estimate of oscillation theta band amplitude for each block within the six conditions were used as parameter weights for subsequent fMRI analysis (see below).

### fMRI data acquisition and analyses

The functional MRI data were recorded with a Siemens 3T Magnetom TIM Trio system with a 12-channel phased-array head coil. BOLD signals contrast was determined by a T2*-weighted echo planar gradient-echo imaging sequence (TR = 2000 msec; TE = 30 msec; FA = 90 degrees; FOV = 192 mm). Each volume consisted of 37 slices acquired parallel to the AC-PC plane (interleaved acquisition; 3 mm with 0.5 mm gap; 3 × 3 mm in-plane resolution). Before the functional runs, a high-resolution T1-weighted sagittal MPRAGE sequence image of the whole brain was acquired (TR = 15 msec; TE = 4.2 msec; FA = 9 degrees; FOV = 256 mm). Functional data were realigned to the first volume acquired and spatially smoothed with a 6.0 mm full-width at half-maximum Gaussian kernel using the AFNI software package [30]. Each functional run was mean-centered and detrended for linear and polynomial trends using linear least squares. Parameter related modulations of the BOLD signal were modeled in a generalized linear model (GLM) framework with independent regressors for task related activity corresponding to each of the 6 tasks of interest (2 sensory modalities X 3 spatial locations), defined for each mini-block of trials of a similar type. In addition to these 6 regressors of the mean BOLD response for each condition, separate regressors were included that varied proportionally with the amplitude in the theta band for each mini-block. The inclusion of these regressors allowed us to determine BOLD activity that scaled linearly with theta amplitude in each condition. Maps of the resulting 12 parameter estimates (beta values) were computed from the GLM from each session and normalized on a subject-by-subject basis to a standard space (MNI) template. Group maps were created by computing a single sample t-test comparing the group mean at each voxel to 0. All reported clusters of activity were corrected for multiple comparisons via Monte Carlo simulations using AFNI’s 3dClustSim function by ensuring that the size of a given cluster of activation was sufficiently large to rule out discovery by chance (corrected α < 0.05). Those significant activity in BOLD would reveals large scale circuits that have mean activity correlated with EEG theta oscillation.

## Results

### Behavior

**Fig 2:**
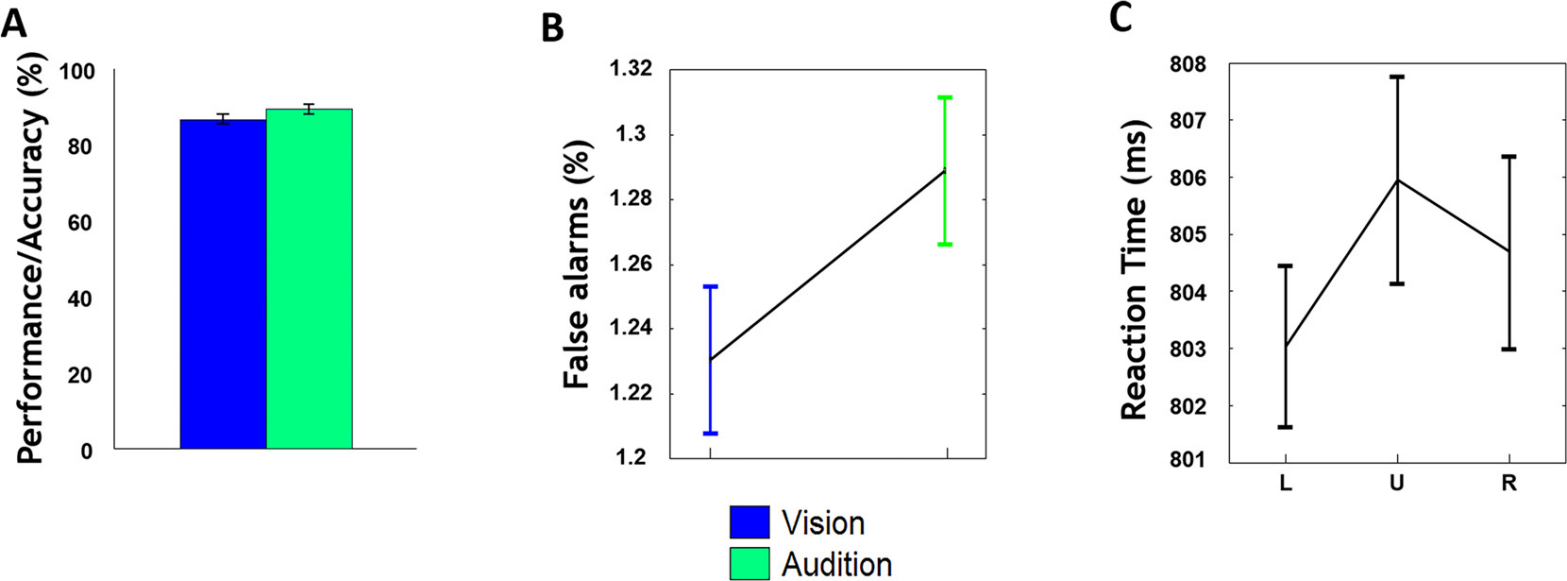
Behavioral performance and the correlation between behavioral reaction time. (A) The performance accuracy during visual and auditory target detection. (B) The false alarms for visual and auditory blocks. (C) The mean RT from combing visual and auditory on three target locations, i.e. left (L) up (U) right (R). Error bars represent, for each condition, ±SE across subjects.

In general, cross-modal visual target detection was faster than cross-modal auditory target detection across all three spatial locations. The mean RT on trials with a visual target (570 ms) was significantly shorter than those with an auditory target (1038 ms) (P < 0.001, two-way ANOVA). The slower cross-modal auditory target detection processing was expected given similar findings using other multi-modal discrimination tasks [31–33]. There was no significant difference in mean accuracy across the three spatial locations (left: 84%; up: 85%; right: 87%) and between visual (84%), auditory (86%) target detection (P = 0.72, two-way ANOVA), see Fig 2A. The mean false alarm (FA) rate during visual attention was not significantly different from that during auditory attention (P = 0.13, two-way ANOVA), see Fig 2B. There was no significant difference in mean RT of vision and audition across three spatial locations of stimuli streams (P = 0.3, one-way ANOVA), see Fig 2C. Given the absence of behavioral differences as a function of spatial location, we collapsed the subsequent fMRI results across spatial position and focused on sensory modality effects irrespective of spatial attention.

### ITC and ERSP

**Fig 3:**
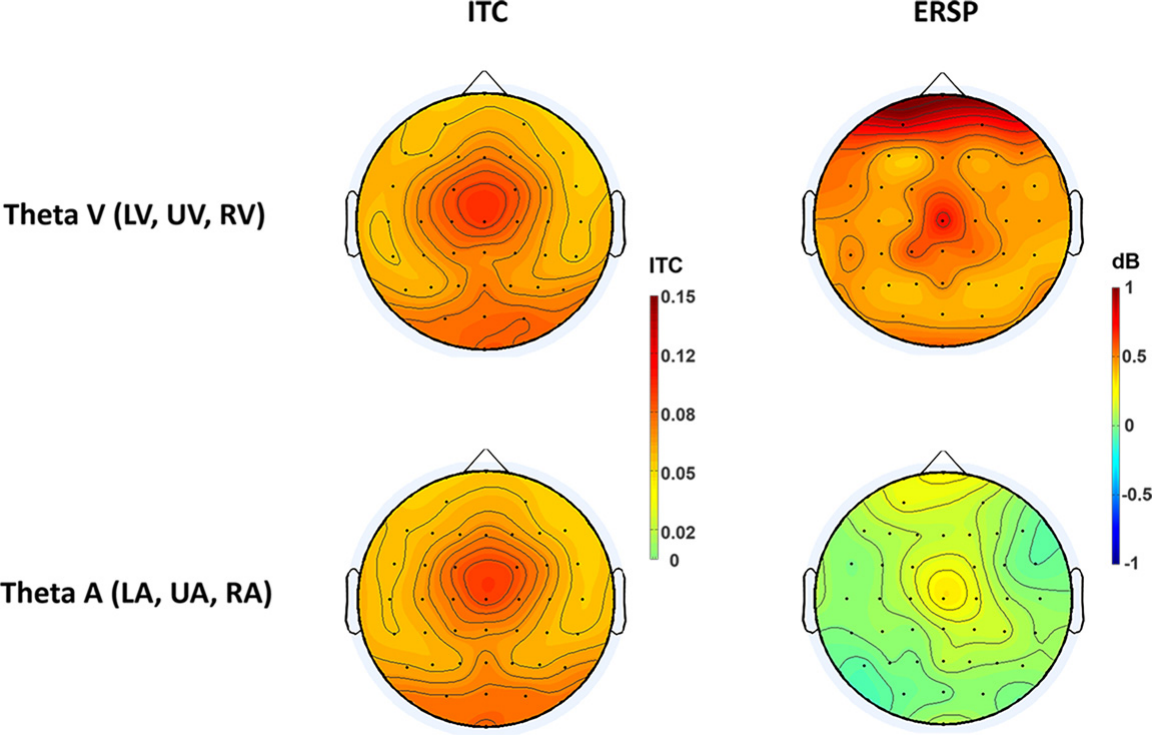
Group-level ITC and ERSP scalp topographies based on theta band oscillation. *Up-left*: Group-level topography of ITC for visual attention. *Up-right*: Group-level topography of ERSP for visual attention. *Down-left*: Group-level topography of ITC for auditory attention. *Down-right*: Group-level topography of ERSP for auditory attention. All results averaged across the three spatial (L, U and R) conditions.

The results of ITC estimated for each trial, collapsed across spatial location, show a phase-locking event happening in the theta band on both visual and auditory tasks and located in central and rostral areas of the scalp, especially in the middle-frontal region. Corresponding results from ERSP show a strong synchronization of power during visual and auditory attention, focused particularly on middle-frontal regions of scalp maps, as shown in Fig 3. These results support our selection of Fz and Cz electrodes for measuring theta power across all six conditions.

### Differences of BOLD activity as a function of cross-modal attention

**Fig 4:**
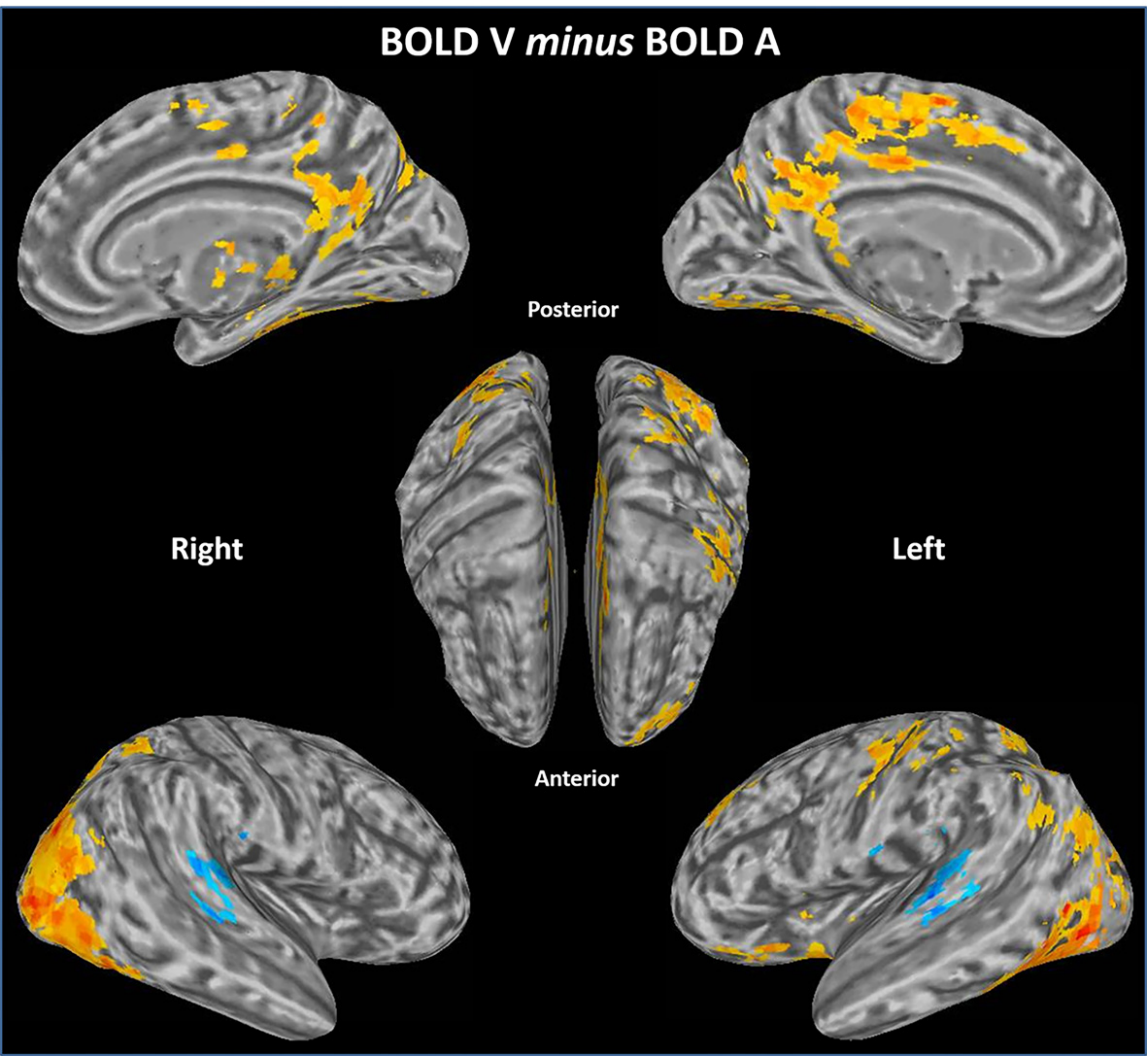
Crossmodal effect of sensory modality on differences of BOLD signal. A direct comparison between BOLD activity from all visual attention blocks was compared with all auditory attention blocks. The auditory cortex (STG) shows significantly greater activity (blue) during auditory attention, while the visual cortex, the regions relevant with DMN (ACC, AG, PCC and Precuneus) and left dorsal frontal parietal network shows significantly greater activity (yellow and red) during the visual attention compared to the auditory task. This was an exploratory analysis, with p<0.05 corrected. Results are collapsed across three spatial locations (L, U and R).

Before considering the interaction of theta amplitude with BOLD activity, we first tested if there was a classic cross-modal differences of BOLD activity as a function of sensory modality. To do this, BOLD activity from all blocks of trials with visual attention was directly compared with blocks of trials with auditory attention (collapsed across spatial locations). This comparison (p<0.05 corrected) was intended to supplement the subsequent interpretation of the effects of theta power on BOLD activity. As shown in Fig 4 and Table 1, when attending to visual stimuli there was significantly greater activity in superior occipital gyrus (SOG), middle occipital gyrus (MOG), inferior occipital gyrus (IOG) and fusiform gyrus (FG), as well as the precuneus, angular gyrus (AG), posterior cingulate cortex (PCC) and anterior cingulate cortex (ACC), and dorsal fronto-parietal network, i.e. middle frontal gyrus (MFG), inferior frontal gyrus (IFG), insular lobe (IL), postcentral gyrus (PostG), supplementary motor area (SMA), superior parietal lobule (SPL), inferior parietal lobule (IPL) (red). When attending to auditory stimuli, there was relatively greater activity (blue) in bilateral superior temporal gyrus (STG). These cross-modal differences in primary sensory areas as a function of sensory modality are consistent with previous studies [34] and demonstrate this effect generalizes to naturalistic stimuli.

**Table 1:** Average MNI coordinates (peak), size and t-value of clusters showing significant Crossmodal effect of sensory modality on differences of BOLD signal (P < 0.05, corrected).

Anatomical Region	Hemisphere	Size	X	y	z	t-value
MOG, IOG, FG, AG, SOG, SPL	R-	2033	-44	78	-4	3.84
IOG, MOG, SPL, IPL	L-	1744	48	69	-6	3.83
SMA, Precuneus, PCC	M-	805	3	3	57	3.70
IFG, IL	L-	297	36	-24	-18	3.72
PostG	L-	215	42	18	42	3.67
STG	L-	162	63	27	9	3.80
MFG	L-	152	30	-33	27	3.65
Thalamus	R-	117	-15	27	0	3.76
ACC	M-	112	-3	-36	15	3.66
STG	R-	109	-66	30	12	3.77

Cluster size is given in voxels (3×3×3). Abbreviations: L-, left; R-, right; M-, middle; MFG, middle frontal gyrus; IFG, inferior frontal gyrus; ACC, anterior cingulate cortex; IL, insula lobe; PostG, postcentral gyrus; SMA, supplementary motor area; STG, superior temporal gyrus; PCC, posterior cingulate cortex; SPL, superior parietal lobule; IPL, inferior parietal lobule; AG, angular gyrus; SOG, superior occipital gyrus; MOG, middle occipital gyrus; IOG, inferior occipital gyrus; FG, fusiform gyrus.

### Supramodal networks—The relationship between theta oscillation and BOLD across all trials

**Fig 5:**
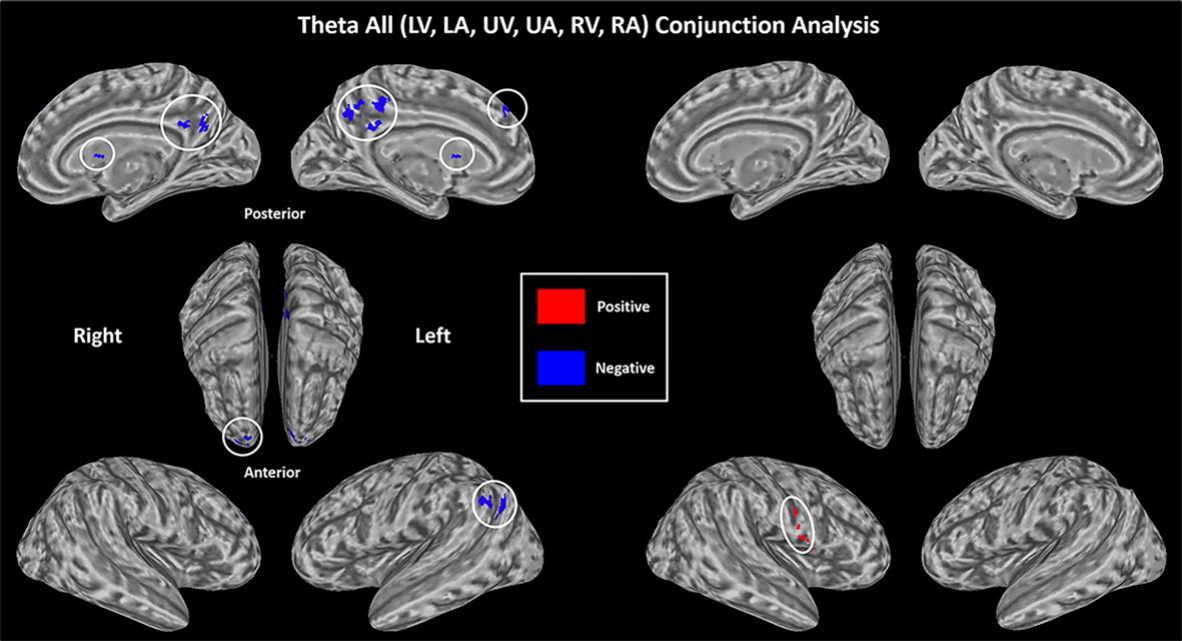
Supramodal correlates of theta amplitude based on conjunction analysis. (p < 0.05, corrected). Results are collapsed across 3 spatial (L, U and R) conditions. There is an overall negative relationship of theta and BOLD in bilateral PCC and CN, left mSFG and AG, right SFG (blue). There is an overall positive relationship of theta and BOLD in right ventral PreG (red).

To identify the likely supramodal sources of theta oscillations, we estimated the relationship between EEG oscillation and BOLD activity for each of the six types of attentional state (2 sensory modalities X 3 spatial locations). We then collapsed across sensory location, resulting in both positive and negative correlations related to either visual or auditory attention. To assess supramodal sources, we calculated the conjunction combining auditory and visual sets of results. As shown in Fig 5 and summarized in Table 2A, the conjunction analysis revealed that theta oscillation significantly displayed a negative linear relationship (P < 0.05, corrected) with BOLD activity in medial segment of the superior frontal gyrus (mSFG), superior frontal gyrus (SFG), AG and PCC, areas of the default mode network (DMN) [35], as well as caudate nucleus (CN). There was an overall positive relationship of theta and BOLD in right ventral precentral gyrus (PreG). That is, weaker the theta power coincided with greater relative BOLD activity.

**Table 2:** Average MNI coordinates (peak), size and t-value of clusters showing significantly correlation between EEG oscillation and BOLD activity (P < 0.05, corrected).

Anatomical Region	Hemisphere	Size	X	y	z	t-value
A, Supramodal correlates based on conjunction analysis						
PCC	M-	128	-2	52	30	
SFG	R-	95	-21	-66	18	
CN	M-	78	6	-9	-3	
mSFG	L-	50	3	-42	36	
PreG	R-	47	-56	-2	18	
AG	L-	38	45	63	27	
B, Theta correlated BOLD—Visual Conditions						
mSFG, ACC, SFG, MidOG	M-	1338	6	-60	18	2.52
M-SMA, SFG, PostG, SPL	L-	930	0	6	57	2.46
PreG, PostG, RO, IL	R-	899	-36	3	42	2.47
AG, IPL	R-	400	-54	60	27	2.36
CN	M-	362	-15	-18	15	2.44
PCC	M-	329	-3	54	30	2.42
C, Theta correlated BOLD—Auditory Conditions						
PreG, RO, STG	R-	840	-33	12	30	2.41
AG, MOG	L-	428	51	60	30	2.38
mSFG, ACC	M-	398	3	-51	15	2.43
Cuneus, PCC	M-	390	6	66	24	2.44

Cluster size is given in voxels (3×3×3). Abbreviations: L-, left; R-, right; M-, middle; SFG, superior frontal gyrus; mSFG, medial segment of the SFG; MidOG, mid orbital gyrus; ACC, anterior cingulate cortex; CN, caudate nucleus; IL, insula lobe; PreG, precentral gyrus; PostG, postcentral gyrus; SMA, supplementary motor area; RO, rolandic operculum; STG, superior temporal gyrus; PCC, posterior cingulate cortex; AG, angular gyrus; SPL, superior parietal lobule; IPL, inferior parietal lobule; MOG, middle occipital gyrus.

### The effects of sensory modality: Modality Specific Networks

**Fig 6:**
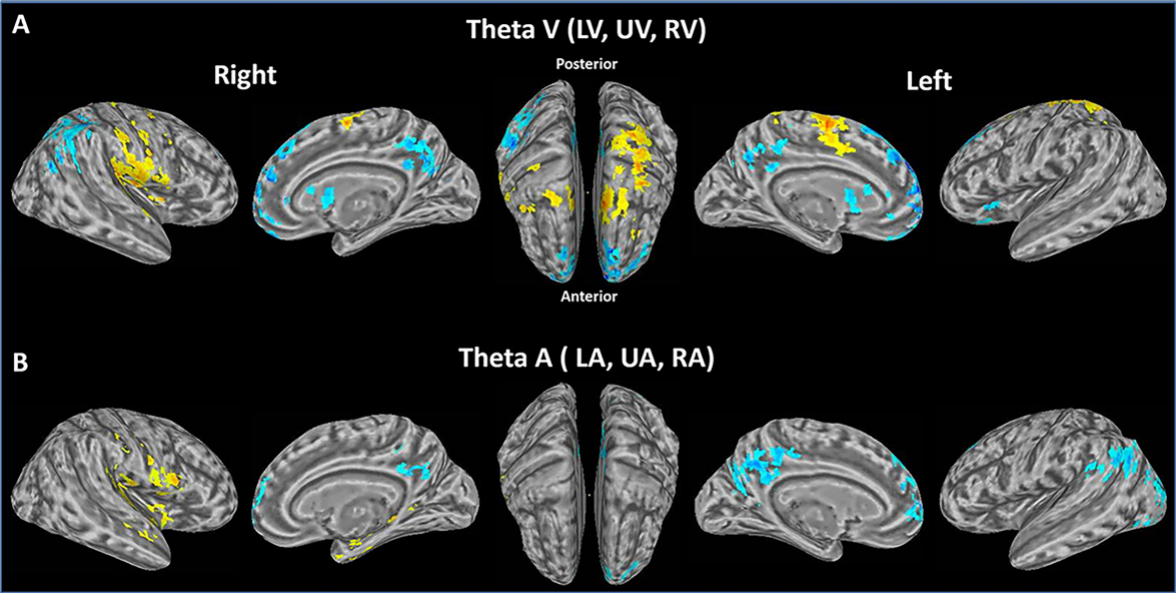
Modality specific interactions between theta and BOLD activity. (p < 0.05, corrected, Red and yellow: positive relationship; blue: negative relationship). (A) Correlates of theta amplitude and BOLD for all visual conditions. Positive relationships are located in bilateral SMA and PostG, left SFG and SPL, right PreG, RO and IL. Negative relationships are localized to mSFG, ACC, SFG, PCC and CN both hemispheres, and right AG and IPL. (B). Correlates of theta amplitude and BOLD for all auditory conditions. Positive relationships are located in right PreG, RO and STG. Negative relationships are localized to mSFG, ACC, Cuneus and PCC both hemispheres, and left AG and MOG.

The previous section demonstrates a strong correlation between theta and BOLD activity in multiple regions in the brain, irrespective of the focus of endogenous attention with respect to either spatial location or sensory modality. We next tested if differences of endogenous attention towards visual or auditory targets would impact the brain network associated with theta activity. Of note, the mean theta amplitude for visual (i.e. LV, UV and RV) and auditory (i.e. LA, UA and RA) attention did not differ (V: 0.567μV; A: 0.527μV; P = 0.168). Thus, any differences as a function of sensory modality could not be attributed to theta amplitude alone. For the visual attention conditions (collapsed across spatial location), there was a significant positive relationship with theta oscillation in bilateral SMA and PostG, left SFG and SPL, right PreG, rolandic operculum (RO) and IL (Fig 6A; Table 2B), consistent with a role of theta in interaction with a set visuomotor regions. Of note, during the visual attention conditions, theta oscillation had a significant negative relationship with BOLD activity in mSFG, ACC, SFG, PCC and CN both hemispheres, and right AG and IPL (Fig 6A; Table 2B). For the auditory attention conditions (collapsed across spatial location), there was a significant positive relationship with theta oscillation in right PreG, RO and STG (Fig 6B; Table 2C). There was a significant negative relationship between theta oscillation and BOLD activity for auditory trials in mSFG, ACC, Cuneus and PCC of both hemispheres, and left AG and MOG (Fig 6B; Table 2C). The left MOG and bilateral cuneus are all associated with visual processing. Other areas, including STG are involved in auditory processing. Together, this indirectly suggests there is also some cross-modal positive influence of theta on cortical areas involved in the relevant sensory modality.

## Discussion

In order to assess the role and source of theta oscillations in cross-modal attention we examined the relationship between theta amplitude averaged over Fz and Cz electrodes and BOLD activity during a cross-modal attention task. Using multiple naturalistic visual and auditory stimuli presented across three spatial locations, we could identify several key EEG-fMRI correlates of endogenous attention. The present results add to the literature by showing that both visual and auditory attention to natural features were significant modulators of theta band activity.

### Supramodal mechanisms

Correlation of theta amplitude with BOLD fMRI showed that it related positively to activity in right ventral precentral gyrus. This area is associated with response selection, prediction and general properties of sensorimotor control, suggesting that the source of theta oscillations changes the gain in this area to facilitate sensorimotor processing. In addition, theta power correlated negatively with activity in precuneus and medial segment of superior frontal gyrus, two brain areas that consistently included the DMN [36]. Recent studies have shown that parametric increases in frontal theta coincide with decreases in BOLD activity in the DMN during visual working memory [19]. We extend this finding to a cross-modal attention task and establish that this is a supramodal effect. One interpretation of this finding is based on the notion that there is a supramodal cognitive control system, likely localized to medial prefrontal cortex and ACC, whose activity is reflected by theta amplitude. Positive interactions with task relevant sensorimotor circuits lead to improved performance. But just as importantly, this circuit is critical for maintaining task "set". Central to this is staying on task and avoiding mind wandering or stimulus independent thought. In this case, one might expect to see an inverse relationship between theta and activity in DMN, which is exactly what we find. The stronger this negative interaction, the more time cognition is “on task”.

### Modality specific processes

Modality specific effects of theta on BOLD activity were particularly apparent in the visual attention condition (Fig 6). Prior studies have supported a role for cognitive control during visual attention that is reflected by frontal theta oscillations with sources in mid frontal regions such as middle cingulate cortex (MCC) and pre-supplemental motor area (Pre-SMA). These are thought to entrain sensory and motor cortices for communication [11]. Furthermore, previous EEG and fMRI studies show that the top-down cognitive modulation from prefrontal cortex could facilitate the neural signal processing in sensorimotor cortex and eventually affect behavioral performance [10,37–39]. Our results show that a positive correlation between theta oscillation and BOLD activity was found in the sensorimotor cortex (PreG, PostG, IL and RO) during cross-modal visual attention (see Fig 6A; Table 2B) similar to other studies of cross-modal visual attention that have been interpreted as the result of top-down cognitive control processes [10]. That positive correlation could be modulated by top-down cognitive control of theta oscillation to facilitate the behavioral response to visual stimuli (see the RT results).

Previous studies show that a fronto-parietal network is involved when subjects engage in frequent switches of task rules [40–42]. As well as, the top-down modulation in task preparatory states during selective attentional control was observed in widely distributed brain regions that form various fronto-parietal networks [43–45]. In addition, the medial prefrontal cortex was phase-lead relative to left parietal particularly on theta band during task preparatory states [45]. Similar to this previous work, the positive correlation between theta oscillation and BOLD activity in our results show that fronto-parietal areas are involved in cross-modal visual attention.

The fact that there are modality associated interactions of theta and the underlying spatial distribution of BOLD activity could be interpreted a number of ways. It could be speculated that cross modal effects are direct, i.e. that auditory attention inhibits visual signal input, while facilitating auditory signal input without the need for supramodal control, and vice versa. In that case, the corresponding interaction of theta-BOLD activities in sensory cortices should be found on both visual and auditory attention conditions, but we have not found them both in the current results. Alternatively, the supramodal involvement of the DMN might imply that cross-modal inhibition is actually dependent on the DMN. The negative correlation of BOLD within the default-mode network (DMN) during multisensory competition has been described previously [10]. Our current results mirror this prior work by also showing a negative relationship between theta amplitude and BOLD activity in DMN that is present for either sensory modality. Others (Huang, et al., 2015) have viewed the involvement of the DMN as causal to the cross-modal inhibition. That is, competition is mediated in part by the DMN. However, since the DMN is not thought to be involved in stimulus dependent task processing or cognitive control, we would argue that it seems unlikely that the cognitive control system is dependent on activity in DMN. A third view, that we advocate here, is that theta oscillations have an influence on both representing the status changes of DMN and separately facilitating the competitive interaction of sensory cortices during auditory attention. We speculate that these theta oscillations may reflect an inhibitory mechanism over the irrelevant sensory modality to avoid processing irrelevant information while at the same time representing involvement of the DMN [11,46,47]. Given the limits of temporal resolution in our fMRI data, we cannot disentangle these alternatives.

### Cross Modal Contributions

To facilitate comparison of our findings with prior studies of endogenous cross-modal attention between visual and auditory modalities we directly compared BOLD activity in these two states (and ignored theta power). As in previous studies, there was a tendency for endogenous attention to enhance relative BOLD activity in relevant sensory processing areas whilst reducing activity in the irrelevant modality. We also noted that regions associated with the DMN (ACC, AG, PCC and Precuneus) likewise showed a relatively higher activity during the visual attention task. In behavioral results, there were shorter RTs for detecting visual targets compared to auditory targets, implying that for the visual attention condition the higher relative activity in DMN might stem from more overall intertrial time spent in stimulus independent thought. Critically, the loci in these results (Fig 4) do not correspond to either supramodal or modality specific differences of theta-BOLD interactions. Thus, theta-related influences on BOLD described in previous sections may not simply be driven by underlying main effects of sensory modality on BOLD activity.

### Methodological concerns

From a behavioral perspective, there was a clear difference of detection speed, with responses to visual targets faster than auditory targets. However, these small differences at the event level are unlikely to influence the estimates of theta band activity or their BOLD correlates because these were based on power estimates over blocks of contiguous trials. Another potential concern was the use of naturalistic, complex stimuli rather than simple letters or symbolic cues. This added complexity might introduce unexpected interactions between attention mechanisms and modality specific systems for decoding complex stimuli. Nevertheless, direct comparisons of BOLD activity during auditory and visual conditions demonstrated the expected cross-modal effect in sensory areas. It is heartening that our results in the visual domain largely replicate prior studies of theta band EEG and confirm that it is possible to generalize studies of attention to naturalistic stimuli.
